# Pre-hospital, in-hospital and post-hospital factors associated with sleep quality among COVID-19 survivors 6 months after hospital discharge: cross-sectional survey in five cities in China

**DOI:** 10.1192/bjo.2021.1008

**Published:** 2021-10-11

**Authors:** Leiwen Fu, Yuan Fang, Dan Luo, Bingyi Wang, Xin Xiao, Yuqing Hu, Niu Ju, Weiran Zheng, Hui Xu, Xue Yang, Paul Shing Fong Chan, Zhijie Xu, Ping Chen, Jiaoling He, Hongqiong Zhu, Huiwen Tang, Dixi Huang, Zhongsi Hong, Xiaojun Ma, Yanrong Hao, Lianying Cai, Jianrong Yang, Shupei Ye, Jianhui Yuan, Yaoqing Chen, Fei Xiao, Zixin Wang, Huachun Zou

**Affiliations:** School of Public Health (Shenzhen), Sun Yat-sen University, Shenzhen, China; Department of Early Childhood Education, The Education University of Hong Kong, Hong Kong SAR, China; School of Public Health (Shenzhen), Sun Yat-sen University, Shenzhen, China; School of Public Health (Shenzhen), Sun Yat-sen University, Shenzhen, China; School of Public Health (Shenzhen), Sun Yat-sen University, Shenzhen, China, and Center for Optometry and Visual Science, the People's Hospital of Guangxi Zhuang Autonomous Region, Nanning, China; School of Public Health (Shenzhen), Sun Yat-sen University, Shenzhen, China; School of Public Health (Shenzhen), Sun Yat-sen University, Shenzhen, China; School of Public Health (Shenzhen), Sun Yat-sen University, Shenzhen, China; School of Public Health (Shenzhen), Sun Yat-sen University, Shenzhen, China; JC School of Public Health and Primary Care, Faculty of Medicine, The Chinese University of Hong Kong, Hong Kong SAR, China; JC School of Public Health and Primary Care, Faculty of Medicine, The Chinese University of Hong Kong, Hong Kong SAR, China; The Fifth Affiliated Hospital of Sun Yat-sen University, Zhuhai, China; The Fifth Affiliated Hospital of Sun Yat-sen University, Zhuhai, China; The Fifth Affiliated Hospital of Sun Yat-sen University, Zhuhai, China; The Fifth Affiliated Hospital of Sun Yat-sen University, Zhuhai, China; The Fifth Affiliated Hospital of Sun Yat-sen University, Zhuhai, China; The Fifth Affiliated Hospital of Sun Yat-sen University, Zhuhai, China; The Fifth Affiliated Hospital of Sun Yat-sen University, Zhuhai, China; Guangdong Provincial People's Hospital, Guangzhou, China; Department of Scientific Research, The People's Hospital of Guangxi Zhuang Autonomous Region, Nanning, China; Department of Education, The People's Hospital of Guangxi Zhuang Autonomous Region, Nanning, China; Department of Hepatobiliary, Pancreas and Spleen Surgery, The People's Hospital of Guangxi Zhuang Autonomous Region, Nanning, China; Department of emergency, SSL Central Hospital of Dongguan City, Dongguan, China; Shenzhen Nanshan District Center for Disease Control and Prevention, Shenzhen, China; School of Public Health (Shenzhen), Sun Yat-sen University, Shenzhen, China; The Fifth Affiliated Hospital of Sun Yat-sen University, Zhuhai, China; JC School of Public Health and Primary Care, Faculty of Medicine, The Chinese University of Hong Kong, Hong Kong SAR, China; School of Public Health (Shenzhen), Sun Yat-sen University, Shenzhen, China; Kirby Institute, University of New South Wales, Sydney, Australia; Shenzhen Center for Disease Control and Prevention, Shenzhen, China; and School of Public Health, Shanghai Jiao Tong University, Shanghai, China

**Keywords:** COVID-19 survivors, sleep quality, pre-hospital factors, in-hospital factors, post-hospital factors, China

## Abstract

**Background:**

Understanding factors associated with post-discharge sleep quality among COVID-19 survivors is important for intervention development.

**Aims:**

This study investigated sleep quality and its correlates among COVID-19 patients 6 months after their most recent hospital discharge.

**Method:**

Healthcare providers at hospitals located in five different Chinese cities contacted adult COVID-19 patients discharged between 1 February and 30 March 2020. A total of 199 eligible patients provided verbal informed consent and completed the interview. Using score on the single-item Sleep Quality Scale as the dependent variable, multiple linear regression models were fitted.

**Results:**

Among all participants, 10.1% reported terrible or poor sleep quality, and 26.6% reported fair sleep quality, 26.1% reported worse sleep quality when comparing their current status with the time before COVID-19, and 33.7% were bothered by a sleeping disorder in the past 2 weeks. After adjusting for significant background characteristics, factors associated with sleep quality included witnessing the suffering (adjusted B = −1.15, 95% CI = −1.70, −0.33) or death (adjusted B = −1.55, 95% CI = −2.62, −0.49) of other COVID-19 patients during hospital stay, depressive symptoms (adjusted B = −0.26, 95% CI = −0.31, −0.20), anxiety symptoms (adjusted B = −0.25, 95% CI = −0.33, −0.17), post-traumatic stress disorders (adjusted B = −0.16, 95% CI = −0.22, −0.10) and social support (adjusted B = 0.07, 95% CI = 0.04, 0.10).

**Conclusions:**

COVID-19 survivors reported poor sleep quality. Interventions and support services to improve sleep quality should be provided to COVID-19 survivors during their hospital stay and after hospital discharge.

Good sleep is part of good quality of life. High-quality and efficient sleep of adequate duration helps to consolidate memory, regulate the immune system and coordinate neuroendocrine functions.^1^ By contrast, poor sleep quality leads to a broad range of adverse outcomes, including cardiovascular diseases, poor mental health status, cognitive impairment and even overall mortality.^2,3^

COVID-19 may negatively affect sleep quality and other mental health outcomes among patients admitted to hospital, patients with mild and asymptomatic cases, and survivors.^4–8^ COVID-19 symptoms and experience during hospital stay are stressors affecting mental health status.^4^ Moreover, treatment for COVID-19 may have adverse effects on mental health and contribute to insomnia.^9^ Furthermore, SARS-Cov-2 might also infect the brain, which could directly cause adverse effects on brain functions and mental health.^5^

Understanding factors associated with sleep quality among COVID-19 survivors after hospital discharge is important for intervention development and service planning to enhance well-being. Cross-sectional studies showed that 29.5% and 26% of COVID-19 survivors in Wuhan, China, were bothered by a sleeping disorder 1 month and 6 months after discharge, respectively (measured by a single item within the Patient Health Questionnaire (PHQ-9): ‘Whether you have trouble falling or staying asleep, or sleeping too much in the past two weeks’ and a self-reported symptom questionnaire: ‘What do you think about your sleeping compared with the status prior to COVID-19’).^6^ A cohort study reported that 26% of COVID-19 survivors suffered from sleep difficulties 6 months after hospital discharge.^10^ Moreover, 40% of COVID-19 survivors in Italy reported insomnia (as measured by the Women's Health Initiative Insomnia Rating Scale).^7^ Only a limited range of factors associated with sleep quality have been reported. Being female, younger and having a history of psychiatric disorder were associated with poor sleep quality.^7^ Previous studies showed that sleep problems among survivors of severe acute respiratory syndrome (SARS) were likely to be persistent; 47%, 50% and 44% of survivors reported difficulty sleeping 3, 6 and 12 months after hospital discharge, respectively.^11^

A systematic review suggested that there were three groups of risk factors for sleep quality among patients after hospital admission and/or critical illness: pre-hospital factors, in-hospital factors and post-hospital factors.^12^ Pre-hospital factors included pre-hospital sleep quality, self-reported concurrent diseases before hospital admission and smoking.^12^ Disease severity at hospital admission, ventilation days, intensive care unit (ICU) admission and length of hospital stay were in-hospital risk factors for sleep quality after hospital discharge.^12^ The main post-hospital factors associated with sleep quality were depression, anxiety and stress.^12^ These factors were considered in this study.

In addition, COVID-19 survivors may have experienced multiple stressors and traumatic events, such as having family members become infected with and/or die of COVID-19, and witnessing the painful symptoms and/or death of other patients.^4^ Corticosteroid therapy is beneficial for modulating the host inflammatory response to COVID-19.^9^ However, sleep disturbance is a commonly cited adverse effect of corticosteroid.^13^ The Chinese government has been providing psychological support for COVID-19 patients and their family members, as well as for people under quarantine, since 8 March 2020.^14^ Screening, prevention and treatment for mental health problems have been pro-actively provided to COVID-19 patients during their hospital stay.^14^ COVID-19 survivors are also encouraged to seek psychological support after hospital discharge. Community healthcare centres are coordinating with psychotherapists and social workers to provide such services to COVID-19 survivors in need.^14^ This study also investigated the association between receiving mental health support services during hospital stay and/or after hospital discharge with post-discharge sleep quality among COVID-19 survivors. Moreover, one study evaluating psychological interventions targeting COVID-19 patients showed that better social support predicted improvement in sleep quality.^15^ Therefore, social support after hospital discharge was also considered by this study. Furthermore, about 7–8% of COVID-19 survivors tested positive again after hospital discharge.^16–18^ However, there was no consensus about the cause of such phenomena.^19,20^ Positive retest results may be another stressor for COVID-19 survivors.

There is a dearth of studies investigating the longer-term impact of COVID-19 on sleep quality. To address these gaps, this study investigated sleep quality among COVID-19 patients 6 months after their most recent hospital discharge. Factors associated with sleep quality were also investigated, including background characteristics and pre-hospital, in-hospital and post-hospital factors.

## Method

### Study design

This was a cross-sectional telephone survey among 199 COVID-19 patients 6 months after their hospital discharge. The study was conducted during August to September 2020.

### Participants and data collection

Participants were recovered adult COVID-19 patients discharged between 1 February and 30 March 2020. The conveniently selected study sites included five hospitals located in five Chinese cities (i.e., Wuhan, Shenzhen, Zhuhai, Dongguan and Nanning) with high, moderate and low COVID-19 case-load. Wuhan is the capital city of Hubei Province, which is most affected by the COVID-19 pandemic in China. Shenzhen, Zhuhai and Dongguan are cities in Guangdong Province, which has the second largest number of COVID-19 cases in China. Nanning is the capital city of Guangxi Province, which has been less affected by the COVID-19 pandemic. As of 9 November 2020, the numbers of recovered COVID-19 patients in these Chinese cities were 46 475 in Wuhan, 72 in Nanning, 468 in Shenzhen, 109 in Zhuhai and 100 in Dongguan.^21^ COVID-19 survivors who were under 18 years old were excluded for the following reasons. First, the measurement of sleep quality (single-item Sleep Quality Scale; SQS) was not validated in a population under 18 years old.^22^ No study used this scale to measure sleep quality among children or adolescents. Second, most of the patients under 18 years old in the records kept by the collaborative hospitals were very young (5–10 years old). It would have been difficult for them to answer our questionnaire, which used relatively complicated psychosocial measurements. Asking their parents to answer the questionnaire would create additional bias.

According to the treatment guidelines in China, recovered COVID-19 patients should be quarantined for 14 days in designated facilities, followed by another 14-day home-based quarantine after hospital discharge. Their contact information is kept by the hospitals for follow-up assessments and services. Recruitment was facilitated by medical staff in the five participating hospitals, who were responsible for follow-up visits and services for recovered COVID-19 patients after hospital discharge. They contacted all discharged COVID-19 patients discharged between 1 February and 30 March 2020 who were listed in their registries. Verbal informed consent was obtained from all participants. Verbal consent was witnessed and formally recorded. The aforementioned medical staff screened prospective participants’ eligibility to join the study, briefed them about the study information and invited them to complete a telephone-based interview. Upon appointment, trained interviewers confirmed participants’ informed consent and conducted the telephone-based interview. Participants were assured that identifiable information would be kept confidential, and that withdrawal from the study would not affect their right to use any treatment services. The interview lasted for about 35 min. No incentive was given to the participants. The authors assert that all procedures contributing to this work comply with the ethical standards of the relevant national and institutional committees on human experimentation and with the Helsinki Declaration of 1975, as revised in 2008. All procedures involving human subjects were approved by the Sun Yat-sen University (Shenzhen) (ref. 2020–031).

Of the 317 recovered COVID-19 patients discharged from these hospitals, 27 were under 18 years old (the scale used in this study was not applicable to these patients), 22 had changed their telephone number and one had died in a car accident. The remaining 267 eligible patients were contacted by the research team; 68 eligible patients refused to participate in the study. A total of 199 eligible patients provided consent and completed the telephone-based interview. The response rate was 74.5% (Wuhan: 31/49, 63.3%; Nanning: 56/72, 77.8%; Shenzhen: 38/50, 76.0%; Zhuhai: 39/51, 76.5%; and Dongguan: 35/45, 77.8%).

### Measures

#### Development of the questionnaire

A panel consisting of one epidemiologist, two public health researchers, a health psychologist and a clinician was formed to develop the questionnaire used in the current study.

#### Background characteristics

Participants were asked to report on sociodemographic characteristics, including age, gender, permanent residency status, highest educational level, relationship status, monthly personal income, employment status and whether they had children.

#### Sleep quality

A single-item SQS was used to measure global sleep quality over a 7 day recall period with a rating ranging from 0 to 10. A higher score indicated better sleep quality.^22^ Cut-off scores of 1, 4, 7, and 10 were used to define poor, fair, good and excellent sleep quality, respectively.^22^ The single-item SQS uses more suitable measurement characteristics to assess sleep quality compared with lengthier sleep questionnaires such as the Pittsburgh Sleep Quality Index (PSQI) and the Morning Questionnaire-insomnia.^22^ This instrument has been demonstrated to be a reliable and valid measure that does not significantly increase the burden on respondents and has been used in a number of published studies,^23–27^ including studies conducted in China.^24^ In addition, participants were asked to compare their current sleep quality with that in the time before COVID-19 (response categories: 1 = got worse, 2 = no change, 3 = got better). One item extracted from the PHQ-9 scale was used to assess whether participants were bothered by sleep disorder: ‘Whether you have trouble falling or staying asleep, or sleeping too much in the past two weeks’ (response categories: not at all, several days, more than half the days and nearly every day).^6^

#### Pre-hospital factors

Participants were asked to report whether they had received a diagnosis of insomnia or other chronic diseases (i.e., hypertension, diabetes mellitus, cancers, and other chronic heart/lung/liver/renal diseases) before COVID-19. Cigarette smoking within 1 year prior to COVID-19 was also recorded.

#### In-hospital factors

Information about severity level of COVID-19 at hospital admission, days in hospital, ICU admission, and the use of invasive ventilation and corticosteroid therapy was extracted from participants’ medical records. Participants were asked whether they had a family member or members with COVID-19 or who had died of COVID-19, and whether they had witnessed the suffering and death of other COVID-19 patients. In addition, participants were asked whether they had received any mental health support services during their hospital stay.

#### Post-hospital factors

Participants reported whether they had received positive SARS-Cov-2 nucleic acid testing results and any mental health support services after hospital discharge.

Depressive symptoms were measured by a validated Chinese version of the PHQ-9 scale, which has been widely used for screening for depression in the Chinese population.^24,28,29^ The Cronbach's alpha of the PHQ-9 was 0.91; one factor was identified by exploratory factor analysis, explaining 59.1% of the total variance.

Generalised anxiety disorder was measured by a validated Chinese version of the Generalized Anxiety Disorder Scale (GAD-7).^30^ The Cronbach's alpha of the GAD-7 Scale was 0.92; one factor was identified by exploratory factor analysis, explaining 68.1% of the total variance.

Post-traumatic stress disorder (PTSD) was measured by the eight-item Post-Traumatic Stress Disorder scale (PTSD-8).^31^ The items correspond to the DSM-IV criteria for PTSD. They are answered on a four-point Likert scale, ranging from 1 (not at all) to 5 (all the time). The summed score provides a score for symptom severity. The internal consistency as measured by Cronbach's alpha (0.89) was good in the current sample.

Four items were used to measure perceived emotional and instrumental support from family members and friends with a rating ranging from 0 (not at all) to 10 (very much). The Perceived Social Support Scale was formed by summing up individual item scores, with higher scores indicating perceived higher social support. The Cronbach's alpha of the Social Support Scale was 0.80; one factor was identified by exploratory factor analysis, explaining 62.8% of the total variance.

### Statistical analysis

The score on the single-item SQS was used as the dependent variable. The associations between independent variables of interest (pre-hospital factors, in-hospital factors and post-hospital factors) and the dependent variable were tested using multivariate linear regression models, after adjusting for significant background characteristics. Adjusted unstandardised coefficients (B) and 95% CIs were reported. SPSS version 24.0 (IBM Corporation) was used to conduct all analyses, with a *P*-value less than 0.05 indicating statistical significance. This was a secondary analysis of a study investigating behaviours and mental health of COVID-19 survivors in China.

## Results

### Background characteristics

Over half of the participants were aged 50 years or younger (63.8%, *n* = 127), female (53.3%, *n* = 106), were married or cohabiting with a partner (81.9%, *n* = 163), had not completed any tertiary education (55.3%, *n* = 110), did not have permanent residency of the city where they lived (73.4%, *n* = 146), had a personal income of less than RMB 6000 (approximately USD 900) per month (74.4%, *n* = 148), did not have a full-time job (59.8%, *n* = 119) and had at least one child (80.4%, *n* = 160) (Table 1).

**Table 1:** Sociodemographic characteristics of participants (*n* = 199)

	*n*	%
Age group (years)		
18–30	33	16.6
31–40	59	29.6
41–50	35	17.6
51–60	33	16.6
>60	39	19.6
Gender		
Male	93	46.7
Female	106	53.3
Relationship status		
Currently single	36	18.1
Married/cohabiting with a partner	163	81.9
Highest education attained		
Junior high or below	53	26.6
Senior high	57	28.6
College and above	86	43.2
Refuse to disclose	3	1.5
Permanent resident of the city		
No	146	73.4
Yes	53	26.6
Monthly personal income (RMB)		
No stable income	71	35.7
<3000	25	12.6
3000–5999	52	26.1
6000–9000	24	12.1
≥10 000	27	13.6
Employment status		
Full-time employment	80	40.2
Freelancer	31	16.1
Student	15	7.5
Retiree	55	27.6
Unemployed	17	8.5
Having at least one child		
No	39	19.6
Yes	160	80.4
City		
Wuhan	31	15.6
Shenzhen	38	19.1
Dongguan	35	17.6
Zhuhai	39	19.6
Nanning	56	28.1

### Sleep quality

Among the participants, 10.1% (*n* = 20) reported terrible or poor sleep quality, and 26.6% (*n* = 53) reported fair sleep quality. The mean score on the single-item SQS was 6.9 (s.d. 2.3). About 30% of the participants (26.1%, *n* = 52) reported worse sleep quality when comparing their current status with the time before COVID-19. Over one-third of the participants had been bothered by a sleeping disorder in the past 2 weeks (33.7%, *n* = 67).

### Pre-hospital, in-hospital and post-hospital factors

Prior to COVID-19, none of the participants had been diagnosed with insomnia, whereas 33.7% (*n* = 67) had other chronic conditions. The most commonly reported chronic conditions were hypertension (*n* = 18, 9.5%) and diabetes mellitus (*n* = 14, 7.0%). One-ninth of the participants (*n* = 22, 11.1%) had smoked in the year prior to COVID-19.

Based on their medical records, the majority of the participants had a moderate level of severity at hospital admission (55.8%, *n* = 111), spent no more than 28 days in hospital (57.3%, *n* = 114), were not admitted to the ICU (97.5%, *n* = 194), and did not undergo invasive ventilation (96.5%, *n* = 192) or corticosteroid therapy (87.9%, *n* = 175). Among the participants, 26.1% (*n* = 52) and 13.1% (*n* = 26) had witnessed the suffering and death of other COVID-19 patients, 42.7% (*n* = 85) had received mental health support during their hospital stay, 44.7% (*n* = 89) reported having at least one family member infected with COVID-19 and 1.5% (*n* = 3) had a family member who had died of COVID-19.

After hospital discharge, 3.5% (*n* = 7) received positive SARS-Cov-2 nucleic acid testing results, and 22.1% (*n* = 44) received mental health support services. The mean scores on the PHQ-9 scale, the GAD-7 scale, the PTSD-8 scale and the Social Support Scale are shown in Table 2.

**Table 2:** Sleep quality and its potential correlates among participants (*n* = 199)

	*n* (%)	Mean (s.d.)
Sleep quality		
Score on the single-item Sleep Quality Scale		6.9 (2.3)
Terrible (0)	3 (1.5)	
Poor (1–3)	17 (8.5)	
Fair (4–6)	53 (26.6)	
Good (7–9)	108 (54.2)	
Excellent (10)	18 (9.0)	
Whether you have trouble falling or staying asleep, or sleeping too much in the past two weeks (an item on the PHQ-9)		
Not at all	132 (66.3)	
Several days	39 (19.6)	
More than half the days	14 (7.0)	
Nearly everyday	14 (7.0)	
Self-reported change in sleep quality comparing current status with the time before COVID-19		
Got worse	52 (26.1)	
No change	145 (72.9)	
Got better	2 (1.0)	
Pre-hospital factors		
Self-reported insomnia diagnosis before COVID-19		
No	199 (100.0)	
Yes	0 (0.0)	
Self-reported chronic conditions other than insomnia before COVID-19		
No	132 (66.3)	
Yes	67 (33.7)	
Smoking before COVID-19		
No	177 (55.3)	
Yes	22 (11.1)	
In-hospital factors		
Severity level of COVID-19 at hospital admission		
Asymptomatic	3 (1.5)	
Mild	42 (21.1)	
Moderate	111 (55.8)	
Severe	24 (12.1)	
Critical	1 (0.5)	
No record	18 (9.0)	
Days in hospital		
≤14	22 (11.1)	
15–28	92 (46.2)	
29–42	49 (24.6)	
>42	11 (5.5)	
No record	25 (12.6)	
ICU admission		
No	194 (97.5)	
Yes	5 (2.5)	
Invasive ventilation		
No	192 (96.5)	
Yes	7 (3.5)	
*Corticosteroid* therapy		
No	175 (87.9)	
Yes	24 (12.1)	
Witnessing the suffering of other COVID-19 patients		
No	147 (73.9)	
Yes	52 (26.1)	
Having a family member infected with COVID-19		
No	110 (55.3)	
Yes	89 (44.7)	
Having a family member die of COVID-19		
No	196 (98.5)	
Yes	3 (1.5)	
Witnessing death of other COVID-19 patients		
No	173 (86.9)	
Yes	26 (13.1)	
Receiving mental health support services during hospital stay		
No	114 (57.3)	
Yes	85 (42.7)	
Post-hospital factors		
Receiving positive SARS-Cov-2 nucleic acid testing results after hospital discharge		
No/did not receive testing after hospital discharge	192 (96.5)	
Yes	7 (3.5)	
Receiving mental health support services after hospital discharge		
No	155 (77.9)	
Yes	44 (22.1)	
Depressive symptoms (score on PHQ-9)^a^		3.2 (5.1)
Anxiety symptoms (score on GAD-7)^b^		2.5 (4.1)
Post-traumatic stress disorder (score on PTSD-8)^c^		28.5 (9.6)
Social Support Scale^d^		11.7 (5.5)

a. PHQ-9, nine items, Cronbach's alpha: 0.91; one factor was identified by exploratory factor analysis, explaining 59.1% of total variance.

b. GAD-7, seven items, Cronbach's alpha: 0.92; one factor was identified by exploratory factor analysis, explaining 68.1% of total variance.

c. PTSD-8, eight items, Cronbach's alpha: 0.89; one factor was identified by exploratory factor analysis, explaining 57.6% of total variance.

d. Social Support Scale, four items, Cronbach's alpha: 0.80; one factor was identified by exploratory factor analysis, explaining 62.8% of total variance.

### Factors associated with sleep quality

Older participants (51–60 years: B = −1.12, 95% CI = −2.21, −0.03, *P* = 0.04; >60 years: B = −1.37, 95% CI = −2.42, −0.32, *P* = 0.01; reference group: 18–30 years) and those who were retired (B = −1.20, 95% CI = −1.97, −0.42, *P* = 0.003; reference group: full-time employment) reported poorer sleep quality. Participants who had a higher monthly income (RMB 3000–5999: B = 0.83, 95% CI = 0.01, 1.65, *P* = 0.048; reference group: no fixed income) and lived in Nanning (B = 1.14, 95% CI = 0.17, 2.10, *P* = 0.02; reference group: Dongguan) reported better sleep quality (Table 3).

**Table 3:** Correlates of sleep quality (*n* = 199)

	Unadjusted B^1^ (95% CI)	*P*-value^a^
Age group (years)		
18–30	Ref.	
31–40	−0.17 (−1.14, 0.79)	0.73
41–50	−0.66 (−1.74, 0.42)	0.23
51–60	−1.12 (−2.21, −0.03)	0.04
>60	−1.37 (−2.42, −0.32)	0.01
Gender		
Male	Ref.	
Female	−0.30 (−0.95, 0.34)	0.35
Relationship status		
Currently single	Ref.	
Married/cohabiting with a partner	−0.60 (−1.43, 0.23)	0.16
Highest education attained		
Junior high or below	Ref.	
Senior high	−0.69 (−1.55, 0.17)	0.11
College and above	−0.05 (−0.84, 0.74)	0.90
Refuse to disclose	0.87 (−1.80, 3.54)	0.52
Permanent resident of the city		
No	Ref.	
Yes	0.05 (−0.68, 0.78)	0.89
Monthly personal income (RMB)		
No fixed income	Ref.	
<3000	0.44 (−0.61, 1.49)	0.41
3000–5999	0.83 (0.01, 1.65)	0.048
6000–9000	0.85 (−0.21, 1.92)	0.12
≥10 000	0.52 (−0.50, 1.54)	0.31
Employment status		
Full-time employment	Ref.	
Freelancer	−0.13 (−1.05, 0.80)	0.79
Student	0.15 (−1.05, 1.40)	0.81
Retired	−1.20 (−1.97, −0.42)	0.003
Unemployed	0.16 (−1.02, 1.35)	0.79
Having children		
No	Ref.	
Yes	−0.64 (−1.44, 0.17)	0.12
Study sites		
Dongguan	Ref.	
Zhuhai	0.97 (−0.70, 2.02)	0.07
Shenzhen	0.37 (−0.68, 1.43)	0.48
Nanning	1.14 (0.17, 2.10)	0.02
Wuhan	0.55 (−0.55, 1.66)	0.33

a. Unadjusted B: unstandardised coefficient.

After controlling for these significant background characteristics, participants who had witnessed the suffering (adjusted B = −1.15, 95% CI = −1.70, −0.33, *P* = 0.006) or death (adjusted B = −1.55, 95% CI = −2.62, −0.49, *P* = 0.04) of other COVID-19 patients had poorer sleep quality. Regarding post-hospital factors, more severe depressive symptoms (adjusted B = −0.26, 95% CI = −0.31, −0.20, *P* < 0.001), anxiety symptoms (adjusted B = −0.25, 95% CI = −0.33, −0.17, *P* < 0.001) and PTSD (adjusted B = −0.16, 95% CI = −0.22, −0.10) were associated with poorer sleep quality, whereas a higher level of social support (adjusted B = 0.07, 95% CI = 0.04, 0.10, *P* < 0.001) was associated with better sleep quality. The association between receiving positive SARS-Cov-2 nucleic acid testing results after hospital discharge and the dependent variable was of marginal statistical significance (adjusted B = −1.56, 95% CI = −3.36, 0.24, *P* = 0.09) (Table 4).

**Table 4:** Correlates of sleep quality among participants (*n* = 199)

	Unadjusted B (95% CI)	*P*-value	Adjusted B (95% CI)	*P-*value
Pre-hospital factors				
Presence of other chronic conditions before COVID-19	−0.36 (−1.04, 0.32)	0.30	−0.09 (−0.81, 0.64)	0.82
Smoking before COVID-19	0.44 (−0.58, 1.46)	0.39	0.26 (−0.78, 1.30)	0.62
In-hospital factors				
Severity level of COVID-19 at hospital admission				
Asymptomatic/mild	Ref	Ref	Ref	Ref
Moderate	−0.05 (−0.81, 0.80)	0.99	−0.39 (−1.30, 0.51)	0.39
Severe/critical	−0.57 (−1.71, 0.55)	0.32	−0.40 (−1.66, 0.85)	0.53
No record	0.25 (−1.02, 1.51)	0.70	0.63 (−0.64, 1.91)	0.33
Days in hospital				
≤14	Ref	Ref	Ref	Ref
15–28	0.26 (−0.82, 1.34)	0.64	0.69 (−0.39, 1.77)	0.21
29–42	0.70 (−0.47, 1.87)	0.24	0.77 (−0.42, 1.96)	0.20
>42	0.46 (−1.22. 2.13)	0.59	1.06 (−0.64, 2.77)	0.22
No record	0.50 (−0.83, 1.82)	0.46	1.26 (−0.09, 2.60)	0.07
ICU admission	−1.56 (−3.60, 0.48)	0.13	−1.43 (−3.45, 0.59)	0.17
Invasive ventilation	−1.25 (−2.99, 0.48)	0.16	−1.01 (−2.85, 0.84)	0.28
*Corticosteroid* therapy	−0.82 (−1.79, 0.16)	0.10	−0.82 (−1.85, 0.21)	0.12
Having a family member infected with COVID-19	−0.17 (−0.81, 0.48)	0.61	−0.37 (−1.06, 0.33)	0.30
Having a family member die of COVID-19	0.75 (−1.88, 3.39)	0.57	0.90 (−1.76, 3.56)	0.51
Witnessing suffering of other COVID-19 patients	−1.10 (−1.81, −0.38)	0.003	−1.15 (−1.697, −0.33)	0.006
Witnessing death of other COVID-19 patients	−1.24 (−2.18, −0.31)	0.01	−1.55 (−2.62, −0.49)	0.04
Receiving mental health support services during hospital stay	−0.30 (−0.95, 0.35)	0.36	−0.25 (−0.91, 0.41)	0.46
Post-hospital factors				
Receiving positive SARS-Cov-2 nucleic acid testing results after hospital discharge	−1.55 (−3.28, 0.18)	0.08	−1.56 (−3.36, 0.24)	0.09
Receiving mental health support services after hospital discharge	0.48 (−0.29, 1.25)	0.22	0.37 (−0.43, 1.18)	0.37
Depressive symptoms (score on PHQ-9)	−0.25 (−0.31, −0.20)	<0.001	−0.26 (−0.31, −0.20)	<0.001
Anxiety symptoms (score on GAD-7)	−0.24 (−0.32, −0.17)	<0.001	−0.25 (−0.33, −0.17)	<0.001
Post-traumatic stress disorder (score on PTSD-8)	−0.17 (−0.22, −0.12)	<0.001	−0.16 (−0.22, −0.10)	<0.001
Social Support Scale	0.08 (0.05, 0.12)	<0.001	0.07 (0.04, 0.10)	<0.001

## Discussion

In our study, the proportion of participants having had sleep disorders in the past 2 weeks (33.7%) was slightly higher than that of a group of Chinese COVID-19 survivors 1 month after hospital discharge (29.2%).^6^ Compared with a previous survey using the same one-item SQS to measure sleep quality among healthy Chinese factory workers in March 2020, the prevalence of terrible or poor sleep quality was much higher in our study (10% *v.* 3.7%).^24^ These findings point to the necessity of interventions and services aimed at improving sleep quality among COVID-19 survivors.

The findings provide some empirical insights that could inform intervention development and service planning, and suggest the need to tailor such interventions and services to specific groups. Older participants were more likely to have poor sleep quality after hospital discharge. Previous studies have shown changes in the sleep cycles of elderly people, for instance, decreased total sleep time, increased time spent falling asleep at sleep onset, sleep fragmentation and daytime sleep.^32^ They should receive more attention in future programmes. Participants without a fixed income were more likely to have poor sleep quality. Although COVID-19 treatment is free in China, hospital admission and subsequent quarantine may affect the work of the survivors and may increase financial difficulties, especially among those with low or no fixed income. Financial difficulties represent a risk factor for sleep quality.^33^ Future programmes should consider providing financial assistance to COVID-19 survivors who have financial difficulties. Participants in Nanning reported better sleep quality than those in the other cities. Compared with the other four study sites, Nanning has been less affected by COVID-19. The COVID-19 situation in the city may be a structural factor influencing people's sleep quality.

In contrast to our hypothesis, pre-hospital factors such as presence of insomnia, other concurrent chronic conditions and smoking were not significantly associated with sleep quality in this study. Witnessing painful symptoms and deaths of other COVID-19 patients were significant in-hospital factors associated with poor sleep quality. These experiences are major psychological traumas for COVID-19 survivors, which may result in PTSD, a mental disorder leading to serious distress and disability.^4,34^ In line with other studies targeting COVID-19 survivors,^7^ our study found a high level of PTSD in this population. PTSD was negatively associated with sleep quality.^12,35^ Moreover, PTSD is long-lasting among survivors of infectious disease outbreaks (e.g., SARS). The median time taken for PTSD to remit is about 36 months for individuals who have sought mental health support services, and can be as long as 64 months for those who have never sought such services.^36^ A meta-analysis suggested that early intervention could lead to durable effects on PTSD severity.^37^ However, as the majority of participants had been discharged from hospitals before the government started providing mental health support services to all COVID-19 patients,^14^ only 42.7% received such services during their hospital stay. It is also unclear whether such mental health support services targeted traumatic experience, as the results showed a non-significant association between in-hospital mental health support service utilisation and sleep quality. In contrast to our hypothesis, ICU admission and the use of invasive ventilation and corticosteroids were not associated with post-discharge sleep quality, probably owing to the very small number of COVID-19 survivors who had such experiences (2.5–12.1%).

Similar to previous findings, depressive symptoms and anxiety symptoms were significant post-hospital factors that were associated with poorer sleep quality.^12^ The associations between depressive and anxiety symptoms and sleep quality were bidirectional. Poor sleep quality might increase the risk of depression and anxiety, whereas presence of depressive symptoms or anxiety symptoms would increase the risk of sleep disturbance.^12,38^ One recent study suggested that sleep is a mediator of the relationship between stress and mental disorders in people infected with SARS-Cov-2.^8^ Such findings highlight the need to provide mental health support services to COVID-19 survivors after hospital discharge. Currently, these mental health support services are provided to COVID-19 survivors upon their request,^14^ and utilisation is relatively low (22.1%). Future programmes should actively reach out to COVID-19 survivors to increase coverage, and the services should be tailored to the needs of COVID-19 survivors. Increasing social support may be a useful strategy to improve post-discharge sleep quality. It is important to provide COVID-19-related health education to the general public and to family members of survivors, so as to reduce unrealistic fears and stigma towards COVID-19 survivors. These approaches may improve social support for COVID-19 survivors. In addition, having a positive SARS-Cov-2 retest result was marginally associated with poor sleep quality. As the cause of this phenomenon is still under investigation,^19,20^ receiving a positive retest result may trigger fear, worry and other negative emotional responses among COVID-19 survivors. In the future, longitudinal studies should be conducted to explore whether COVID-19 survivors with sleep disorders have worse recovery than those without.

This study had the strengths of recruiting survivors in multiple Chinese cities and considering pre-hospital, in-hospital and post-hospital factors. Although several studies have applied the single-item SQS in various Chinese populations,^24,39^ there has been no validation study conducted in a Chinese population. This was a weakness of this study. The study had some other limitations. First, there was no appropriate control group (e.g., patients who were admitted to hospital for reasons other than COVID-19 during the same period). Second, as compared with longer sleep questionnaires (e.g., PSQI), the single-item SQS was not able to cover important information such as sleep latency, sleep duration or habitual sleep efficiency. It merely reflected subjective sleep quality. Similar to some published cross-sectional studies, we measured self-reported changes in sleep quality before and after the COVID-19 outbreak.^6^ However, such measurements are susceptible to strong recall bias. Therefore, we were not able to conclude that our participants had a decrease in sleep quality. Third, the sample size of this study was relatively small. The effect sizes of the non-significant associations were quite small, and the non-significance cannot be fully explained by the relatively small sample size. Fourth, COVID-19 survivors were recruited in five Chinese cities; generalisations should be made cautiously to other geographic locations in China. Fifth, we were not able to collect information from survivors who refused to participate in the study. Therefore, we could compare characteristics between participants and those who refused to complete the survey and hence could not estimate the impact of refusals on study outcomes. Selection bias existed. Sixth, parts of the data were self-reported, and verification was not feasible. Recall bias may have occurred, and we were not able to estimate the impact of recall bias on study outcomes. Moreover, we did not collect information about history of psychiatric disorders, which is a significant factor in post-discharge sleep quality. Furthermore, this was a cross-sectional study and could not establish causal relationships.

Among the COVID-19 survivors, a significant proportion were bothered by sleep disorders, and many reported terrible or poor sleep quality 6 months after hospital discharge. Witnessing the suffering or death of other COVID-19 patients was a significant in-hospital factor associated with poor sleep quality after hospital discharge. Depressive symptoms, anxiety symptoms, PTSD and social support were significant post-hospital factors influencing sleep quality. Interventions and support services to improve sleep quality should be provided to COVID-19 survivors.

## Data Availability

The data that support the findings of this study are available from the corresponding author, H.Z., upon reasonable request.
