# Pre-hospital, in-hospital and post-hospital factors associated with sleep quality among COVID-19 survivors 6 months after hospital discharge: cross-sectional survey in five cities in China – CORRIGENDUM

**DOI:** 10.1192/bjo.2021.1051

**Published:** 2021-11-18

**Authors:** Leiwen Fu, Yuan Fang, Dan Luo, Bingyi Wang, Xin Xiao, Yuqing Hu, Niu Ju, Weiran Zheng, Hui Xu, Xue Yang, Paul Shing Fong Chan, Zhijie Xu, Ping Chen, Jiaoling He, Hongqiong Zhu, Huiwen Tang, Dixi Huang, Zhongsi Hong, Xiaojun Ma, Yanrong Hao, Lianying Cai, Jianrong Yang, Shupei Ye, Jianhui Yuan, Yao-Qing Chen, Fei Xiao, Zixin Wang, Huachun Zou

**Keywords:** COVID-19 survivors, sleep quality, pre-hospital factors, in-hospital factors, post-hospital factors, China

In the original publication,^1^ an error was made regarding an author's name. Yao-Qing Chen was incorrectly written as Yaoqing Chen. This has now been corrected in both the online PDF and HTML versions of this article. The authors apologise for this error.
